# Gout in the Chest Misdiagnosed as Ankylosing Spondylitis

**DOI:** 10.3389/fmed.2020.582444

**Published:** 2020-10-14

**Authors:** Wenjing Xue, Shengkai Zhang, Qinqin Wang, Wenzhong Que, Shanghua Xu

**Affiliations:** ^1^Department of Rheumatology, The Affiliated Nanping First Hospital of Fujian Medical University, Nanping, China; ^2^Department of Cardiology, The Affiliated Nanping First Hospital of Fujian Medical University, Nanping, China

**Keywords:** gout, hyperuricemia, ankylosing spondylitis, dual energy CT (DECT), case report (source: MeSH NLM)

## Abstract

Gout is a crystal-related joint disease caused by single sodium urate deposition in the joints or in soft tissues. In recent years, the incidence of gout has increased, but cases of urate crystals deposited in the chest–ribs are rare. Here, we describe a 39-year-old man who complained of frequent pain and a feeling of tightness in chest–ribs and was misdiagnosed as ankylosing spondylitis. In addition, treatment with non-steroidal anti-inflammatory drugs (NSAIDs) and etanercept for 6 months showed no improvement, which confirmed the misdiagnosis. After physical examination, blood examination, and dual-energy CT examination, the patient was diagnosed with gout and received 50 mg benzbromarone once a day with treatment of low serum uric acid. In conclusion, gout in the chest and ribs is an unusual manifestation and has rarely been reported in the literature. This case highlights an important but overlooked history of hyperuricemia in the diagnosis, and dual-energy CT is the preferred method for differential diagnosis of chest–ribs gout.

## Case Presentation

**Figure 1:**
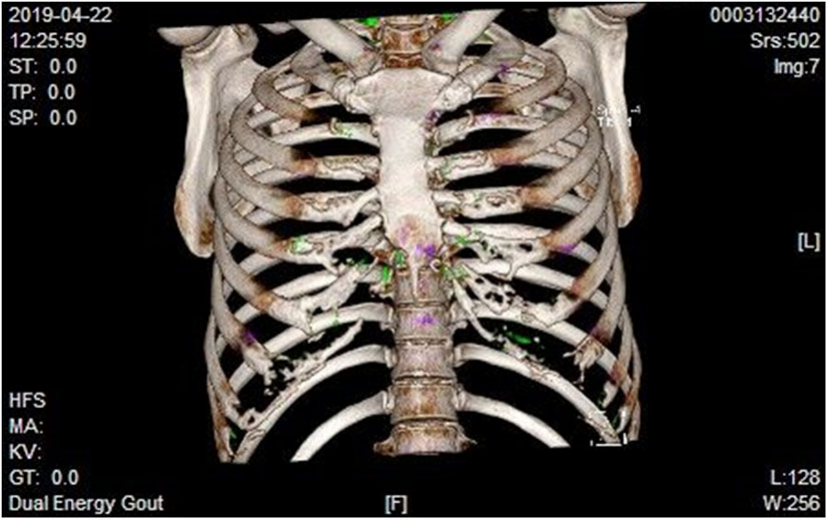
Dual-energy CT examination of the chest. Urate crystal deposition is indicated in green.

We report a case of a 39-year-old man whose main complaints were chest pain and tightness in the past 2 years. He had been taking non-steroidal anti-inflammatory drugs (NSAIDs) orally over the past year, but the relief was not obvious. Six months ago, he was diagnosed as ankylosing spondylitis (AS) and treated with etanercept, but the symptoms have not been alleviated, and chest and back discomfort was worse. He has no other history of disease, although hyperuricemia has been reported in previous hematological tests. The patient has no history of trauma, uveitis, psoriasis, inflammatory bowel disease, or pain in other joints. There was no obvious abnormality in the chest, heart, and abdomen. Thoracic mobility was normal. There was no movement restriction of the spine. He had tenderness of the fourth to seventh thoracic vertebrae but no tenderness in the sacroiliac joints. The straight-leg elevation test was negative. The results of the blood test showed high uric acid (9.9 mg/dL, reference value: 3.4–7.0 mg/dL) and positive human leukocyte antigen B27 (HLA-B27). Erythrocyte sedimentation rate, C-reactive protein (CRP), blood tumor markers, and other blood tests were normal. Electrocardiogram, cardiac color Doppler ultrasound, full abdominal color Doppler ultrasound, and chest CT excluded heart disease and tumor. Both CT and MR of sacroiliac joints suggested mild degeneration of bilateral sacroiliac joint and no bone edema. When the patient visited our department, we considered the possibility of uric acid crystal deposition and performed dual-energy CT (DECT) examination of the chest. Urate crystal deposition was found in the bilateral chest; costal joint; costal cartilage; third left, first bilateral, and second costal vertebrae; costal head joint; first and second lateral transverse process; and left upper scapula (Figure 1). According to his medical history, physical examination, laboratory examination, and DECT examination, urate crystals may be the main culprit.

## Discussion

Gout is a metabolic rheumatism that is common in middle-aged and elderly men. The main manifestations are crisis of joint pain, swelling, tenderness, and elevated skin temperature. In the vast majority of patients, there is hyperuricemia, and urate is mainly deposited in the joints and periarticular tissue. Deposit in facet joints is a very rare event (1). Pain and tightness in the chest and ribs may indicate diseases such as inflammation of the ribs, inflammatory low back pain, heart disease, or chest tumors, but gout should also be considered, despite its rare occurrence.

**Figure 2:**
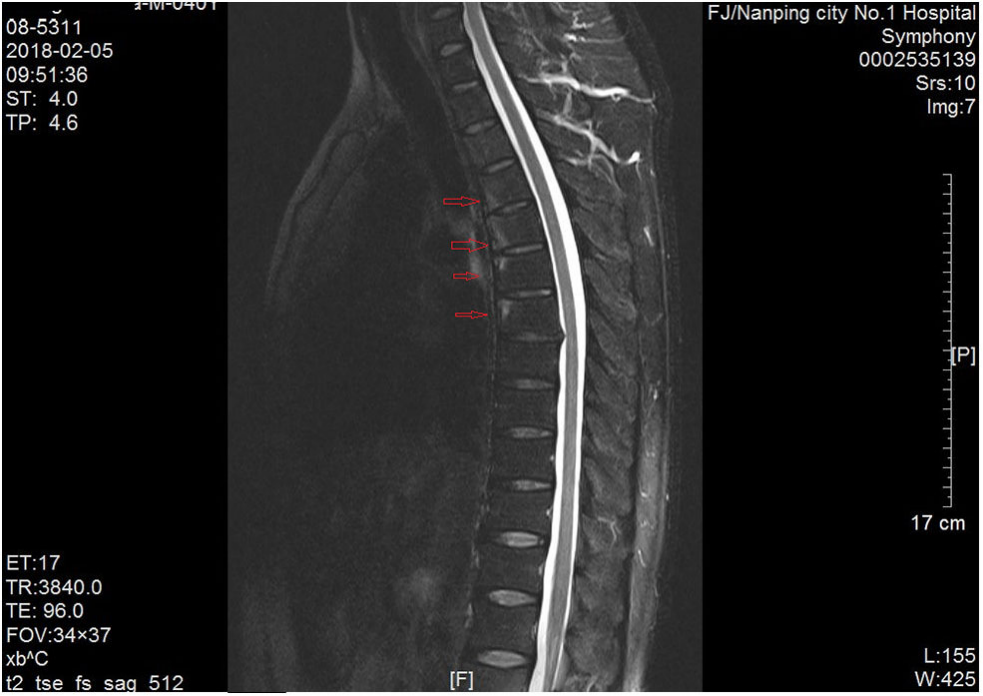
MRI examination of thoracic vertebrae. Abnormal signal of T4–T7 was indicated by a red arrow.

This is an interesting case of a man who has experienced recurrent chest and back pain and chest tightness over the past 2 years. Electrocardiogram, cardiac color Doppler ultrasound, total abdominal color Doppler ultrasound, lung CT, and related blood tests were performed to exclude heart diseases, tumors, and inflammatory diseases. Both CT and MR of thoracic vertebrae showed abnormal signal changes of the thoracic vertebrae in T4–T7, so it is necessary to consider the possibility of AS. However, no sacroiliitis was found in CT and MR. ESR and CRP were normal. In addition, he has no family history of psoriasis and AS. The patient consulted many doctors and was misdiagnosed as AS because of HLA-B27(+) and abnormal signal changes of T4–T7 (Figure 2).

AS is a progressive chronic inflammatory disease that affects the axial skeleton, leading to structural damage and dysfunction. Clinical manifestations of AS usually begin in late adolescence or early adulthood and rarely after the age of 40. The characteristic clinical symptoms of AS are inflammatory back pain, IBP, and morning stiffness, but they are often not well-recognized at the first visit (2). In addition, although 90% of AS patients are HLA-B27 positive, in our clinical practice, we often encounter the situation of HLA-B27-positive patients being misdiagnosed as AS.

Our case was HLA-B27 positive, with repeated chest and back pain for 2 years. However, the most common site of AS is the sacroiliac joint. We performed CT and MRI of the sacroiliac joint and found no sacroiliitis. ESR and CRP were normal. He has no family history of psoriasis and AS. Therefore, the diagnosis of AS is not accurate. In addition, treatment with NSAID and etanercept for 6 months showed no improvement, which confirmed the misdiagnosis of AS. He had a history of hyperuricemia, and the patient was never treated seriously. His serum uric acid level provided an important clue to the diagnosis of the disease. We conducted chest dual-energy CT and found that the thoracic vertebrae and chest–ribs were covered with green urate crystals. In conclusion, despite all new diagnostic tools, establishing a correct diagnosis of gout remains one of the daily challenges of clinical rheumatologists.

## Data Availability Statement

All datasets presented in this study are included in the article/supplementary material.

## Ethics Statement

The studies involving human participants were reviewed and approved by Fujian Medical University. The patients/participants provided their written informed consent to participate in this study. Written informed consent was obtained from the individual(s) for the publication of any potentially identifiable images or data included in this article.

## Author Contributions

WX designed the study. SZ, QW, WQ, and SX performed the study. All authors contributed to the article and approved the submitted version.

## Conflict of Interest

The authors declare that the research was conducted in the absence of any commercial or financial relationships that could be construed as a potential conflict of interest.
